# Cognitive and emotional responses to viewing mummies in an Egyptian museum

**DOI:** 10.3389/fnins.2025.1614268

**Published:** 2026-01-09

**Authors:** M. Iosa, V. Scaramozzino, M. Benente, V. Minucciani, M. Franzò

**Affiliations:** 1 Department of Psychology, Sapienza University, Rome, Italy; 2 SmArt Lab, IRCCS Fondazione Santa Lucia, Rome, Italy; 3 Department of Architecture and Design, Politecnico of Turin, Turin, Italy

**Keywords:** archaeology, cognitive workload, cultural heritage, emotion, mummies, museum, neuroaesthetics

## Abstract

**Introduction:**

A recent subfield of neuropsychology is the study of people’s reactions to visiting a museum and observing artworks. However, museums do not only contain artworks or archeological finds, and some of them exhibit human remains, such as mummies. A growing debate concerns the ethical issues of such exhibitions, but the psychological and physiological reactions of visitors when viewing mummies have not yet been measured.

**Methods:**

In this study, 33 subjects (40.3 ± 14.4 years old) participated in two experiments conducted at the Egyptian Museum of Turin (Italy). In the first experiment, they were asked to observe an empty sarcophagus, an opened sarcophagus with a mummy inside, and an open sarcophagus with the mummy placed between the cover and the coffin of the sarcophagus. Subjects wore an electroencephalographic (EEG) system on their heads, electrodes on their fingers to measure skin conductance levels (SCL) and wore eye-tracking glasses. In the second experiment, they visited the room of the “Three Sisters” with two completely bandaged mummies and one partially unbandaged. The indices extracted from EEG and SCL signals were compared before and after they noticed the partially unbandaged mummy.

**Results:**

Cognitive workload was found to increase due to the presence of the mummies in the first experiment, whereas an increase in emotional arousal (SCL) was observed in the second experiment after participants saw that partially unbandaged mummy.

**Discussion:**

The presence of mummies increased the emotional engagement, but it was not an effect specific leading to negative emotions.

## 1 Introduction

Neuroaesthetics is one of the emerging disciplines within Neuroscience, focusing on the study of neurophysiological and psychological processes underlying the creation of and appreciation of artworks. It integrates the investigation of perception, creativity, and aesthetic feelings of pleasure and judgments elicited by art with research on the functional specialization of brain regions (Zeki et al., 2020).

The availability of wearable devices, such as portable electroencephalographs and heart rate monitors and galvanic skin response electrodes, has allowed researchers to conduct experiments outside the laboratory and directly in museums. This is important because an artistic experience may depend on the location, the situation, and the cultural context in which it is experienced, especially in environments rich in artistic objects, such as museums (Carbon, 2019). Previous studies have investigated participants’ neurophysiological parameters in front of paintings, sculptures, or other artworks (Modica et al., 2016; Babiloni et al., 2013, 2014, 2015). Babiloni et al. (2014) showed that when the observer is positioned in front of the face of a statue (the Moses of San Pietro in Vincoli in Rome), there is higher emotional arousal, measured by skin conductance level, compared to the condition in which the observer and the statue are not face to face. The researchers also measured an approach-withdrawal index, related to the asymmetry in electroencephalographic activity, finding that a more distant point of view implied greater cortical appreciation, probably related to the possibility of observing a general view of the whole sculpture. This study highlighted the importance of what the subject is observing in an artwork in eliciting different physiological reactions. However, museums also contain other types of “objects” which are conventionally less investigated, such as urns and other archaeological finds.

Recently, neurophysiological parameters have been measured in subjects observing the famous Sarcophagus of the Spouses at the National Etruscan Museum in Rome, Italy. It is a tomb effigy considered one of the masterpieces of Etruscan craftsmanship, although it was not created to be displayed as an artistic object, but rather as a funerary item to be placed in a tomb (Giorgi et al., 2023). In this study, the authors adopted materials and methods similar to those of Babiloni et al. (2014) to compare the observation of the real sarcophagus in the museum with the viewing of its virtual reality reproduction in a laboratory. Emotional arousal, assessed by combining data extracted from galvanic skin response and heart rate variability, was higher for the original artwork, but the approach–withdrawal index was increased in the virtual environment. Using electroencephalographic signals, they also measured cognitive workload, recording a higher level during observation in the museum compared to the virtual reproduction of the sarcophagus (Giorgi et al., 2023).

Other important archaeological finds exhibited in some museums are mummies, the most famous of which are the Egyptian mummies. There are also ethical considerations to take into account regarding the exhibition and investigation of mummies, as they are the preserved bodies of deceased humans (Kaufman and Rühli, 2010; Gill-Frerking, 2021). There is an intrinsic dehumanization of these bodies, and museums could be interested in understanding the psychological reactions of visitors to mummies.

A French study based on questionnaires showed that exposure of human tissues, organs or cadavers raises considerable ethical issues (Charlier et al., 2014). A Greek study showed that 46.3% of visitors have moral concerns about seeing human remains, and 28% believe that the exhibits may affect visitors’ mental health, with 26.6% citing a violation of human dignity as the reason (Raikos et al., 2012).

To the best of our knowledge, there are no investigations of the neurophysiological reactions of visitors when they view a mummy in a museum setting. This information could be significant for Museum curators and organizers as well as for researchers interested in the field of cultural well-being and psychophysiological effects of human remains on museum visitors.

In this study, we conducted two experiments to record in the Egyptian Museum of Turin (Italy) the neurophysiological responses of museums visitors when faced with mummies. In the first experiment, we investigated the impact of watching a sarcophagus without or with a mummy placed inside or outside; while, in the second experiment, the impact of watching a partially unbandaged mummy compared to completely bandaged mummies was examined. The aim was to understand the cognitive and emotional responses of the visitors when viewing different expositions of mummies.

## 2 Materials and methods

### 2.1 Participants and location

Thirty-three subjects participated in the following two experiments (age: 40.3 ± 14.4 years, 23 females). They were enrolled among the visitors of the museum. All the participants had already visited the Egyptian Museum of Turin, so, despite possible changes in the museum installations, it was not the first visit to the museum for any of them. Hence, we could consider this sample as referring to the population of museum goers. During both experiments, participants wore a portable electroencephalographic system, an ear-clip for assessing heart rate, electrodes on the fingers of the non-dominant hand for measuring skin conductance level (SCL), and eye-tracking glasses.

All participants signed informed consent to participate in the study. The study was approved by the Ethics Committee for Translational Research of Sapienza University (Project Neuro-Museum, protocol 204/2024). The experiments were conducted at the Egyptian Museum of Turin, Italy, on days when the museum was closed to the public, ensuring that no other visitors were present.

### 2.2 Experiment 1

In the first experiment, participants were positioned by the researcher in front of one of three sarcophagi with their eyes closed at a distance of 1 m. They were then asked to stand still, open their eyes upon the researcher’s signal, and visually explore the sarcophagus placed in front of them for 1 min (measured by the researchers). Participants were instructed not to walk, speak, or touch the sarcophagi during the experiment, except in cases of discomfort, in which they could request a rest or to stop the experiment, however, no participant made such a request.

**Figure 1:**
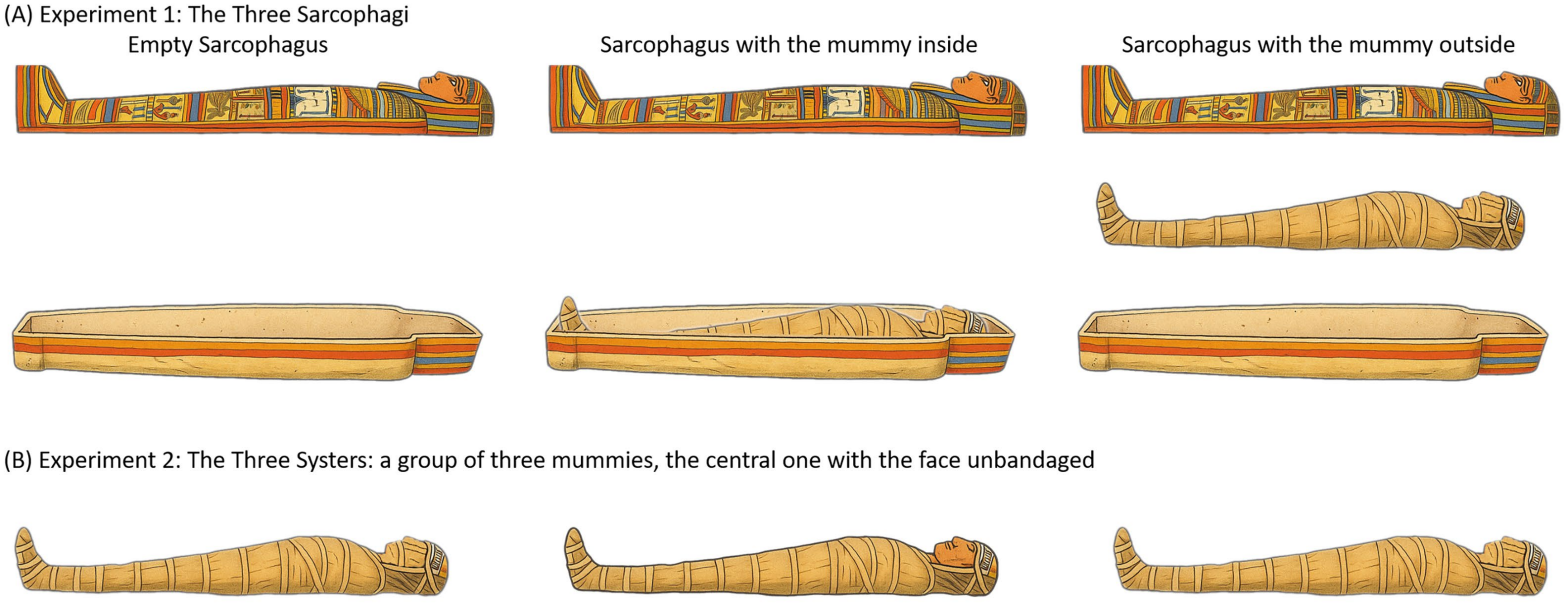
Representation of the conditions in the Experiment 1 (**A**, above) and Experiment 2 (**B**, below).

Participants were tested under three different conditions corresponding to the three different sarcophagi. One of the sarcophagi was empty (with the cover placed at a certain distance from the sarcophagus), one sarcophagus contained a mummy (with the cover placed above at a proper distance to allow the view of the mummy), and the third was open with the mummy placed between the base and the cover (as shown in Figure 1A, real photos of sarcophagi and mummies could be found at^1^: for seeing their positions). The order of presentation of the three sarcophagi was randomized among participants. Specifically, each participant was placed in front of each of the three types of expositions following a randomized list in which each subject was assigned a different order of presentation of the three conditions. The shape and decorations of the three sarcophagi were very similar in shape and decoration.

Then, participants completed a questionnaire about their emotional feelings in each of the three different conditions, in which they rated 15 emotions rated on a Visual Analogue Scale (VAS) ranging from 0 (not at all) to 100 (maximum intensity) (Aitken, 1969; Bond and Lader, 1974).

### 2.3 Experiment 2

The second experiment was performed in another room of the Egyptian Museum, the so-called “Three Sisters room,” which contains three mummies, two bandaged and one partially unbandaged (with her face uncovered), their three sarcophagi and other archaeological finds (Figure 1B).

Participants were simply instructed to freely walk around the room and explore the installation. Using eye tracking, we identified if and when participants viewed the unbandaged part of one of the mummies. Neurophysiological indices were averaged across two time windows: before (condition PRE) and after (condition POST) this moment.

Since the three mummies were close to each other in the same room, it was not possible to administer a questionnaire to distinguish between responses to the bandaged and unbandaged mummies in the second experiment.

### 2.4 Neurophysiological measures and indices

We adopted the same measurement protocol used by Cartocci et al. (2021), and subsequently adopted by Giorgi et al. (2024), which we briefly summarize below.

Before proceeding with the experimental task, baseline neurophysiological signals were recorded. Participants were instructed to close their eyes for 1 min to record cortical signals for computing the Individual Alpha Frequency (Klimesch, 1999). Subsequently, they were asked to fixate on a plain wall for 1 min to record their resting-state activity, which served as a reference condition in the analysis.

The electroencephalographic device was the Mindtooth Touch headset (Brain Products GmbH, Gilching, Germany—BrainSigns srl. Rome, Italy), which is easy to fit and comfortable. It presents eight Ag/AgCl electrodes with water-based sponges placed according to the 10–10 International System (AFz, AF3, AF4, AF7, AF8, Pz, P3 and P4), with reference and ground electrodes, placed on the mastoids. The device has been validated and is capable of recording EEG signals with high accuracy (Sciaraffa et al., 2022). The sampling frequency was 125 Hz. A 50 Hz notch filter was applied to remove mainline power interference. EEG recordings were also band-pass filtered (high-pass filter cut-off frequency: 2 Hz; low-pass filter cut-off frequency: 40 Hz). Then, the same artifact removal protocol used by Giorgi et al. (2024) was applied.

From the EEG signal, following previous studies (Cartocci et al., 2021; Chiarella et al., 2022; Giorgi et al., 2023), a Cognitive Workload Index (CWL) and an Approach–Withdrawal Index (AW) were computed.

The CWL represented the mental resources allocated to visually processing the stimulus and was computed as the average EEG signal in the Theta band across the frontal brain region. Theta activity in frontal regions is associated with an increased cognitive workload demand during task execution (Sciaraffa et al., 2019; Schultze-Kraft et al., 2016; Davidson, 2004; Modica et al., 2017). This increased Theta activity reflects heightened activation of the prefrontal cortex, which is involved in higher-level cognitive processes. The index was normalized using the median and median absolute deviation of the baseline.

The Approach–Withdrawal Index was derived from the brain’s asymmetric response to pleasant and unpleasant stimuli. Asymmetry in the Alpha band of frontal EEG signals has been reported in the scientific literature as being related to motivation to withdraw from a stimulus and the tendency to reject and avoid it (Di Flumeri et al., 2016; Davidson, 1995; Davidson, 2004; Vecchiato et al., 2011). The Alpha band activity in the right and left frontal regions was calculated by averaging the values of the right and left electrodes, respectively. A higher index value represents a greater motivation to approach the stimulus (Cartocci et al., 2021). This index was normalized using the median and median absolute deviation of the baseline.

Electrodermal activity was recorded using the Shimmer3 GSR + Unit (Shimmer Sensing, Dublin, Ireland) placed on the second and third fingers of the participant’s non-dominant hand. The sampling frequency was 64 Hz. The signal recorded by the Shimmer was first low-pass filtered with a cut-off frequency of 1 Hz and then decomposed to estimate the tonic component (Skin Conductance Level—SCL) (Braithwaite et al., 2013). SCL is the slow-changing component of the electrodermal signal and is mostly related to the participant’s overall arousal (Benedek and Kaernbach, 2010). The Shimmer3 + device was also used to collect a photoplethysmographic signal (PPG) using a PPG-to-HR ear clip, filtered using a 5th-order Butterworth bandpass filter (1–1 Hz and 1–4 Hz, respectively), to derive a heart rate (HR) estimation (Goovaerts et al., 1976; Giorgi et al., 2023).

Wearable glasses for eye-tracking (Tobii Pro Glasses 2, Tobii, Stockholm, Sweden) were used for identifying the moment when participants viewed the partially unbandaged mummy in Experiment 2.

### 2.5 Statistical analysis

Data are reported as means and standard deviations. In Experiment 1, Repeated Measures Analysis of Variance (RM-ANOVA) was conducted to compare the three conditions. For the RM-ANOVA, a sphericity check of the data was performed, and the Greenhouse–Geisser correction was applied when sphericity was violated. Tukey’s correction was applied to *p*-values for post-hoc analyses. Partial eta squared was computed to assess the effect size in the RM-ANOVA. Pearson’s coefficient (R) was used to assess correlations between neurophysiological indices. For the VAS-scores of the questionnaire, given that these variables were ordinal and not-normally distributed, Friedman’s analysis was used. In Experiment 2, the Wilcoxon rank test was used to compare the neurophysiological indices within subject before and after participants viewed for the first time the unbandaged face of the mummy. The alpha significance level was set at 0.05 for all the performed analyses.

## 3 Results

### 3.1 Experiment 1

**Figure 2:**
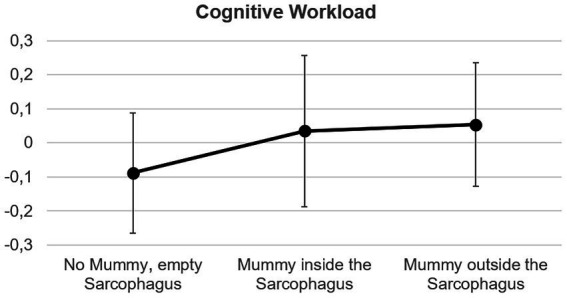
Mean ± standard deviation of the indices cognitive workload in the Experiment 1.

In Experiment 1, significant differences between the three conditions were observed in terms of CWL (Figure 2 and Table 1). Post-hoc analysis revealed that cognitive workload was lower in the condition without the mummy (*p* = 0.003, for both comparisons involving the conditions with the mummy), whereas cognitive workload was not statistically different between the two conditions with the mummy (*p* = 0.876). No differences were observed in the other neurophysiological indices.

**Table 1:** Analysis of variance of neurophysiological indices computed in the Experiment 1: columns report the *F* (degrees of freedom), the *p*-value (Greenhouse–Geisser correction was applied to *p*-value of Heart Rate because of a violation of sphericity of data on this variable) and the effect size measured in terms of partial eta squared.

Neurophysiological index	*F*(2, 64)	*p*	Partial eta squared
Cognitive workload	8.496	0.001^*^	0.210
Approach–withdrawal	1.078	0.346	0.033
Skin conductance level	0.326	0.723	0.010
Heart rate	0.149	0.804	0.005

*p < 0.05.

No significant correlations were found among the four parameters assessed in the three conditions, except in the condition with the mummy placed outside the sarcophagus, where heart rate was significantly and negatively correlated with the AW index (*R* = −0.554, *p* = 0.001).

VAS scores of the questionnaire indicated that the most strongly reported emotions were interest, aesthetic enjoyment, and wonder, with no statistically significant differences among the three conditions (Table 2). Significant differences were observed for boredom, which was lower when a mummy was present (regardless of whether it was inside or outside the sarcophagus), melancholy (which was lower when the mummy was inside the sarcophagus), and pleasure (which was also significantly reduced in that condition).

**Table 2:** Mean ± standard deviation of feelings VAS score in the three conditions (the last two columns report the results of Friedman analysis, ^*^p < 0.05).

Feelings	Without Mummy	Mummy inside	Mummy outside	*χ* ^2^	*p*
Boredom	18 ± 24	5 ± 13	4 ± 8	26.8	<0.001*
Restlessness	11 ± 17	9 ± 16	15 ± 20	3.6	0.168
Melancholy	17 ± 18	7 ± 8	17 ± 21	7.5	0.023*
Tiredness	9 ± 12	7 ± 11	5 ± 7	4.3	0.116
Sadness	9 ± 13	8 ± 11	13 ± 18	0.8	0.680
Amusement	9 ± 14	13 ± 18	11 ± 17	1.0	0.594
Excitement	20 ± 22	24 ± 32	21 ± 27	4.2	0.120
Wellbeing	24 ± 25	26 ± 30	28 ± 29	1.3	0.535
Wonder	52 ± 25	50 ± 30	53 ± 25	2.1	0.344
Pleasure	34 ± 30	30 ± 32	35 ± 33	7.6	0.022*
Aesthetic enjoyment	53 ± 31	52 ± 34	51 ± 33	0.4	0.822
Interest	71 ± 27	73 ± 26	74 ± 24	4.8	0.090
Disappointment	9 ± 18	3 ± 4	4 ± 6	5.4	0.066
Disgust	3 ± 8	3 ± 4	2 ± 4	1.2	0.538
Irritation	2 ± 3	2 ± 3	2 ± 3	2.3	0.311

### 3.2 Experiment 2

In Experiment 2, the data of eye tracking device showed that 23 participants had at least one fixation point located on the unbandaged face of the central mummy, whereas 10 subjects (42 ± 14 years old, 6 females) had not any fixation on that, meaning that they did not directly watch the unbandaged face of that mummy throughout the experiment. The data of these 23 participants are reported in Table 3 divided between pre- and post- watching the unbandaged mummy. A statistically significant increase in SCL was observed after participants viewed the partially unbandaged mummy. The reduction in CWL was not statistically significant, and the approach-withdrawal index showed minimal variation. Both their values, pre and post watching the unbandaged mummy, were not significantly different from the relevant values of subjects who did not watch the unbandaged mummy (*p* > 0.05). No statistically significant correlations were found between neurophysiological indices.

**Table 3:** Mean ± standard deviation and relevant analysis of variance of neurophysiological indices computed in the Experiment 2.

Neurophysiological index	Pre	Post	*P*
Cognitive workload	0.48 ± 0.42	0.33 ± 0.34	0.201
Approach–withdrawal	0.01 ± 0.08	−0.01 ± 0.05	0.447
Skin conductance level	0.63 ± 0.27	0.71 ± 0.25	0.002^*^

In the last column, the *p*-value (^*^ < 0.05) was obtained by Wilcoxon rank test.

## 4 Discussion

The aim of this study was to investigate how the viewing of a mummy may impact the psycho-physiological state of a museum visitor. The two experiments reported interesting results. In the first experiment, the presence of a (bandaged) mummy, regardless of whether it was placed inside or outside the sarcophagus, had a statistically significant impact on cognitive workload, but not on emotional parameters. Cognitive workload is an index of the mental resources allocated to visually process the stimulus (Sciaraffa et al., 2019; Cartocci et al., 2021), and hence, the presence of the mummy could increase the complexity of the scene to be visually explored. In general, it is well known that aesthetic experiences in a museum elicit widespread brain arousal, since there is no single cerebral area exclusively devoted to aesthetics, but rather a set of cortical and subcortical areas forming a network of circuits involved in the sensory-motor processing of knowledge, significance, and emotions related to the observed stimulus (Starr, 2013; Pelowski et al., 2017). Our findings can also be interpreted as indicating that the vision of the mummy may elicit more thoughts in the visitor, suggesting an increase in cognitive workload not only due to the higher complexity of the scene when the mummy is present (in both conditions, inside and outside the sarcophagus), but also due to its reflective impact compared to when the sarcophagus is empty. When visitors are asked to comment on the experience of viewing a mummy, most of them report reflective thoughts or negative comments, while others express mixed feelings or positive remarks (Squires et al., 2024). To clarify this point in our study, the answers to the questionnaire are helpful, as they highlight that the presence of the mummy mainly reduced the visitors’ boredom (*p* < 0.001). Previous studies showed that observing a real sarcophagus induces a higher cognitive workload than watching its virtual reproduction (De Giorgi et al., 2023). The presence of the mummy further increased the observers’ cognitive workload.

The Approach–Withdrawal Index, which can be considered an index of implicit liking (or disliking) of a wide variety of stimuli (Briesemeister et al., 2013), did not show significant differences among the conditions presented in our study. However, when the mummy was outside the sarcophagus, participants with a higher motivation to mentally withdraw from the mummy (those with a lower AW value) were those also having a higher heart rate (as shown by the statistically significant correlation). Interestingly, when our participants saw the mummy inside the sarcophagus, they reported on the PANAS scale feeling less pleasure (*p* < 0.05) and less melancholy (*p* < 0.05) than in the other two conditions (when the mummy was outside the sarcophagus or when the mummy was absent). A possible interpretation of our results is that this condition could be considered the most “natural” for a mummy, but also the one most clearly associated with death. It could thus reflect a sort of respect for the exposition of the mummy in her sarcophagus. This interpretation is in line with the emotional and respectful approach to exhibit ancient Egyptian dead suggested by Weiss (2024).

While the mummies were bandaged in the first experiment, in the second experiment the museum exhibits one of the three mummies with the face unbandaged. When the visitor looked at her face for the first time, a significant change in the SCL occurred, with a significant increase. There was also a reduction in cognitive workload, but it was neither significant nor correlated with the skin conductance. SCL is an indicator of the global arousal of the visitor (Benedek and Kaernbach, 2010; Braithwaite et al., 2013), elicited by the unbandaged mummy. Interestingly, some subjects, consciously or not, avoided directly looking at the partially unveiled mummy. The *p*-values > 0.05 showed that no statistically significant differences were present between the values of neurophysiological indices of the subjects who did not look at the uncovered face of the mummy, and the other participants before they watched it.

These neurophysiological results were also supported by the psychological questionnaire administered in the first experiment: the lowest level of boredom was recorded in front of the mummies. Despite not being statistically significant, the absence of the mummy was associated with increased disappointment and tiredness. The reduction of tiredness and fatigue has previously been reported as it can be reduced in the presence of artistic stimuli with respect to other types of stimuli, a phenomenon called Michelangelo effect (Iosa et al., 2021).

In our study, the recording of EEG, PPG, and electrodermal activity was used, which represents the most advanced approach in neuroaesthetics (Mazzacane et al., 2020). The recording in a real museum setting was fundamental, because it is well known that the atmosphere within a museum can affect the aesthetic experience by generating an aura that may even exceed the effects of a single object (Dorrian, 2014). However, the choice to collect data in the museum resulted in some limitations of the study mainly related to the acquisition of variables that could not be controlled as they would be in a laboratory. For example, in the second experiment, the proximity of the three mummies did not allow for a clear differentiation of the participants’ reactions to the bandaged and unbandaged mummies, preventing the administration of specific questionnaires for the different bandage conditions. Furthermore, the sample size was limited, though sufficient to highlight some statistically significant results. Nevertheless, the reduced sample size may affect the generalizability of our findings, as well as the fact that the experiments were conducted in two rooms of a single museum. Our group was homogeneous in terms of experience, as all participants had already visited the museum, but this may have limited their physiological reactions, which could be more pronounced in people visiting the museum for the first time. Even the exhibition modalities could not be controlled by the researchers, as they depended on the decisions made by the museum personnel. However, this is the first study investigating the effect of exposing bandaged and unbandaged mummies on cognitive and emotional parameters of museum visitors. Despite the above limitations, our findings can be of great interest, providing a new neuropsychological approach to the exhibition of human remains in museums. Future studies, conducted on wider samples and involving other museums as well, could also record pupil dilation from eye-tracking data to clarify the affective involvement of participants during the observation of different stimuli in the museum.

In conclusion, the exhibition of mummies did not increase emotions like disappointment, disgust, or irritation. The Approach–Withdrawal Index was not affected by the presence of the mummy, either inside or outside the sarcophagus. Visitors reported a lower level of boredom and their cognitive workload increased in the presence of the mummies. Emotional arousal increased when they observed the unbandaged mummy.

## Data Availability

The raw data supporting the conclusions of this article will be made available by the authors, without undue reservation.

## Footnotes

1
https://www.museoegizio.it/en/discover/human-remains/
